# Systematic evaluation and meta-analysis of Flunarizine Hydrochloride combined with traditional Chinese medicine decoction in the treatment of migraine headaches

**DOI:** 10.1016/j.clinsp.2024.100431

**Published:** 2024-07-03

**Authors:** Dan Fan, Wei Leng, Liqin Zhang

**Affiliations:** Department of Encephalopathy, The Affiliated Hospital to Changchun University of Chinese Medicine, Changchun, Jilin, China

**Keywords:** Flunarizine Hydrochloride, Traditional Chinese medicine decoction, Migraine, Chinese medicine symptom score, Meta-analysis

## Abstract

**Objectives:**

To systematically evaluate the efficacy and superiority of Flunarizine Hydrochloride when combined with Traditional Chinese Medicine (TCM) Decoctions in treating migraine headaches.

**Method:**

The authors conducted a comprehensive search for clinical Randomized Controlled Trials (RCTs) investigating the combination of Flunarizine Hydrochloride with Chinese herbal decoctions in treating migraines. The databases searched included CNKI, VIP, Wanfang, PubMed, WOI, Cochrane Library, and Embase, covering the period from January 1, 2019, to November 10, 2023. Two independent researchers meticulously screened, extracted, and assessed the relevant data, employing the Revman 5.3 software for meta-analysis.

**Results:**

The meta-analysis revealed that, in comparison to Flunarizine Hydrochloride used in isolation, the combination with Chinese herbal decoctions markedly enhanced the effective rate (RR = 1.26, 95 % CI [1.18, 1.34], *p* < 0.0001). Moreover, significant improvements were observed in the TCM symptom score (MD = 4.97, 95 % CI [-6.74, -3.19], *p* < 0.00001). The observation group demonstrated a statistically significant improvement in endothelin levels compared to the control group (I^2^ = 85 %, MD = -13.66, 95 % CI [-17.87, -9.45], p = 0.0001). The observation group showed a significant reduction in NRS scores compared to the control group, indicating better outcomes (I^2^ = 95 %, MD = -2.11, 95 % CI [-3.09, -1.12], *p* < 0.0001). The observation group was superior to the control group in terms of the reduction in the number of episodes (I^2^ = 63 %, MD = -1.16, 95 % CI [-1.45, -0.87], p = 0.007).

**Conclusions:**

The confluence of Flunarizine Hydrochloride with traditional Chinese medicine decoctions in treating migraine patients demonstrated substantial clinical efficacy and improvement in TCM symptom score over the use of Flunarizine Hydrochloride alone.

## Highlights

• Flunarizine Hydrochloride combined with TCM notably enhances migraine management.
• Flunarizine Hydrochloride and TCM improve TCM scores, endothelin, NRS and VAS scores.
• Flunarizine Hydrochloride combined with TCM reduces headache frequency and duration.
• Standardized protocols are crucial to understand TCM's role in migraine management better.

## Introduction

Migraine represents a multifaceted polygenic disorder, characterized as a recurrent headache with neurovascular pathophysiological features. Its onset is typically marked by recurring unilateral or bilateral throbbing headaches,^1^ predominantly on the hemianopia side of the head, accompanied by symptoms such as photophobia, phonophobia, nausea, and vomiting. As per the International Classification of Headache Disorders, the third edition, migraines are delineated by a minimum of five attacks and a duration ranging from 4 to 72 h.

Epidemiological studies have illuminated that approximately 125 million individuals globally suffer from migraines, manifesting a male-to-female ratio of 3:1.^2^ The World Health Organization has classified migraine as the sixth most disabling disease globally and the foremost disabling neurological condition. The prevalence of migraine, paralleling that of tetraplegia, psychiatric disorders, and dementia, has placed the clinical management of this ailment under the spotlight, particularly due to its profound impact on patients' lives and cognitive functioning.

Contemporary treatment modalities for migraine encompass a spectrum of approaches, including pharmacotherapy, acupuncture, behavioral therapy, and lifestyle modifications like adequate sleep and exercise. Among these, pharmacological interventions, such as Flunarizine Hydrochloride, are predominantly favored by patients. However, the prolonged use of such medications is often marred by adverse reactions and a recurrence of symptoms following cessation.

In recent years, Traditional Chinese Medicine (TCM) has emerged as an efficacious alternative in migraine management, with decoctions like Chuanxiong Cha Tiao San, XueFu ZhuYu, Sanpian, and Mahuang Fuzi Xixin showing promising results. Scholarly articles have posited that Chinese herbs could serve as potential therapeutic agents in migraine relief, attributable to a myriad of pharmacological factors.^3^

This paper seeks to assimilate and critically assess domestic clinical research literature from the past five years, focusing on the amalgamation of Chinese herbal decoctions with Flunarizine Hydrochloride in treating migraines. The objective is to systematically summarize and evaluate these findings, thereby providing a foundational reference for the clinical application of this combined treatment approach in migraine therapy.

## Data and methods

### *Literature inclusion criteria*

#### Study population

Patients clinically diagnosed with migraine.

#### Intervention

The intervention for the observation group was a combination of herbal decoction and Flunarizine Hydrochloride treatment. The control group received alternative therapies.

#### Outcome indices

The evaluation parameters included the effective rate, recurrence rate, TCM symptom score, endothelin levels, NRS (Numerical Rating Scale) score, VAS (Visual Analogue Scale) score, number of headache episodes, and duration of headaches.

#### Type of study

Only Randomized Controlled Trials (RCTs) examining the efficacy of Chinese herbal decoctions combined with Flunarizine Hydrochloride in treating migraine patients were considered.

### *Literature exclusion criteria*

Excluded from this analysis were non-randomized controlled trial types such as reviews, literature studies, experience summaries, theoretical discussions; animal studies; duplicated publications; studies combining treatment of other diseases; trials where the intervention was not specifically Chinese herbal decoction combined with Flunarizine Hydrochloride in migraine treatment; trials where the intervention combined Chinese herbal decoction with Flunarizine Hydrochloride and other therapies; randomized controlled trials; case reports; and clinical studies with missing outcome data. This meta-analysis was conducted in accordance with the PRISMA guidelines.

### *Literature search*

A comprehensive search was conducted in electronic databases including CNKI, CQVIP, Wanfang, and PubMed. The search utilized the keywords “Traditional Chinese Medicine Decoction Combined Flunarizine Hydrochloride, Cephalagra, Migraine, Randomized Controlled, Flunarizine, Sibelium, Traditional Chinese Medicine” in Chinese, and “Traditional Chinese Medicine Decoction Combined with Flunarizine Hydrochloride, Migraine, Randomized Controlled, Migraine Disorder, Flunarizine” in English. The time frame for this search spanned from January 2019 to November 10, 2023.

### *Data extraction and quality assessment*

Two researchers independently and objectively conducted the literature search, screening, and data extraction, adhering to the predefined inclusion and exclusion criteria. Emphasis was placed on retaining the authenticity of the original data. A pre-designed data extraction form was employed, capturing details such as the authors, year of publication, sample size, method of random allocation, intervention approach, study termination, and instances of participant drop-out, among others. Discrepancies were resolved through consultation with a third researcher.

The quality assessment involved evaluating the risk of bias, encompassing selection bias (creation of randomized sequences, concealment of allocation), implementation bias (blinding of participants and researchers), detection bias (blinding of outcome assessments), dropout bias (completeness of outcome data), reporting bias (selective reporting), and other potential biases. This evaluation was guided by the assessment tools outlined in the second edition of The Cochrane Handbook.

### *Statistical methods*

The Revman 5.3 software was utilized for statistical analysis, adhering to the principles of experimental medicine. Continuous variables, such as TCM symptom score, endothelin levels, NRS scores, VAS scores, number of headache episodes, and headache duration, were synthesized using the Weighted Mean Difference (WMD). Heterogeneity was assessed through the combined effect and 95 % Confidence Interval (95 % CI), employing the Q-test and the I^2^ test. Data from both groups were amalgamated using a fixed-effect model when p ≥ 0.01 and I^2^ <50 %, indicating statistical homogeneity. Conversely, high heterogeneity warranted the use of a random-effects model. Subgroup and sensitivity analyses were conducted to examine the impact of grouping factors on outcomes and to investigate potential sources of heterogeneity from a statistical standpoint. Publication bias risk was evaluated using funnel plot analysis.

## Results

### *Results of literature screening*

**Fig. 1:**
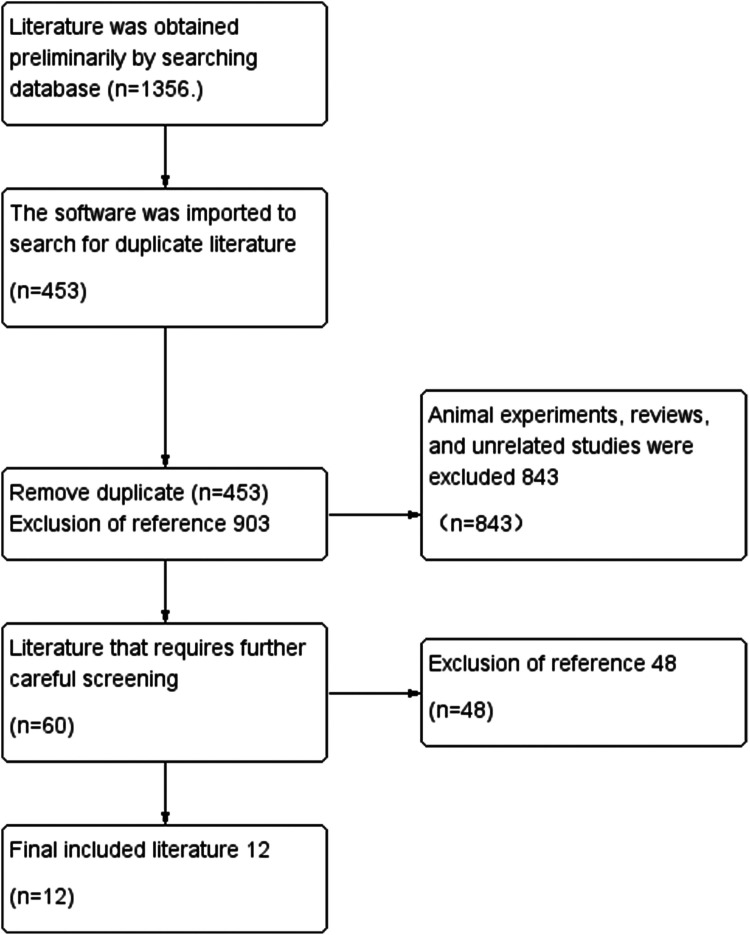
Flow diagram for the selection of the literature.

In the process of sourcing relevant literature from CNKI, CQVIP, WanFang, PubMed, WOI, Cochrane Library, and Embase databases, a total of 1,356 articles were initially identified. Upon removing 453 duplicates, the remaining 903 articles underwent a preliminary screening based on titles and abstracts. This review led to the exclusion of 843 articles due to reasons such as literature reviews, animal studies, theoretical discussions, interventions combined with other therapies, the use of proprietary Chinese medicines, and irrelevance to the research topic. Subsequently, a meticulous evaluation of the full texts of the remaining 60 articles was conducted, resulting in the exclusion of an additional 48 articles. These articles were primarily omitted because 20 of them dealt with migraine in conjunction with other diseases, and 28 did not meet the grouping conditions of the study. Ultimately, 12 articles were selected for inclusion in this meta-analysis (Fig. 1).

### *Basic characteristics of the included studies*

The 12 included studies encompassed a collective sample of 973 migraine patients. The specifics of these studies, including sample size, methodology, and other pertinent details, are delineated in Table 1.

**Table 1:** Included studies.

Included studies	Sample size (T/C)	Intervention		Course of treatment	Outcome indicators
		Observation group	Control group		
Hou Yanping 2022^4^	31/31	XueFu ZhuYu decoction + Flunarizine Hydrochloride	Flunarizine Hydrochloride	4 weeks	①③④⑤⑥
Liu Chang 2019^5^	40/40	SanPain decoction + Flunarizine Hydrochloride	Flunarizine Hydrochloride	4 weeks	①⑧
Wu Yifeng 2022^6^	44/44	SanPain decoction + Flunarizine Hydrochloride	Flunarizine Hydrochloride	January	①②③⑦⑨
Sun Xiaoliang 2022^7^	67/50	ZiYi ZhiXuan decoction + Flunarizine Hydrochloride	Flunarizine Hydrochloride	30 days	⑧⑩
Cui Jinyuan 2022^8^	54/54	XiongZhiShiGao decoction + Flunarizine Hydrochloride	Flunarizine Hydrochloride	4 weeks	①③⑤⑪⑪
Li Jibin 2019^9^	45/45	MahuangFuzi Xixin decoction + Flunarizine Hydrochloride	Flunarizine Hydrochloride	January	①⑦⑧
Wang Bengwei 2022^10^	30/30	MahuangFuzi Xixin decoction + Flunarizine Hydrochloride	Flunarizine Hydrochloride	15 days	①⑤⑪⑫
Wang Zhao 2021^11^	36/36	SanPain decoction + Flunarizine Hydrochloride	Flunarizine Hydrochloride	4 weeks	①⑧⑫
Guo Zhen 2019^12^	36/36	XueFu ZhuYu decoction + Flunarizine Hydrochloride	Flunarizine Hydrochloride	15 days	①②③④
Jin Xiao 2020^13^	40/40	SanPian decoction + Flunarizine Hydrochloride	Flunarizine Hydrochloride	14 days	①⑥ ⑧
Chen Qing 2021^14^	42/42	ZiYin ZhiXuan decoction + Flunarizine Hydrochloride	Flunarizine Hydrochloride	4 weeks	①⑧⑩
Long Sheng 2020^15^	30/30	XiongZhiShiGao decoction + Flunarizine Hydrochloride	Flunarizine Hydrochloride	4 weeks	⑤⑥

Note: T, Represents the group treated with the drug combined with cognitive behavioral therapy; C, Denotes the control group.

① Effective rate; ② Recurrence rate; ③ Endothelin level; ④ Haemodynamics; ⑤ VAS score; ⑥ NRS score; ⑦ 5-HT level; ⑧ TCM symptom score; ⑨ Plasma procalcitonin; ⑩ DHI score; ⑪ Duration of headache; ⑫ Number of headache attacks.

### *Methodological quality evaluation*

Among the 12 studies included in the meta-analysis:

Randomization: Nine studies detailed the use of a randomized numeric table method for allocation, classifying them as low-risk in terms of randomized allocation. However, one study that allocated participants according to the order of consultation was evaluated as high risk.

Blinding: Challenges in implementing blinding during the intervention were noted. Only two studies reported the implementation of blinding. One study was assessed as high risk and another as low risk in the blinded evaluation category. The remaining studies were categorized as having unclear risk due to the absence of blinding details.

Incomplete Data: One study was assessed as high risk due to incomplete data. This was attributed to the loss of two participants during the study - one due to interruption by other illnesses and another due to poor adherence.

**Fig. 2:**
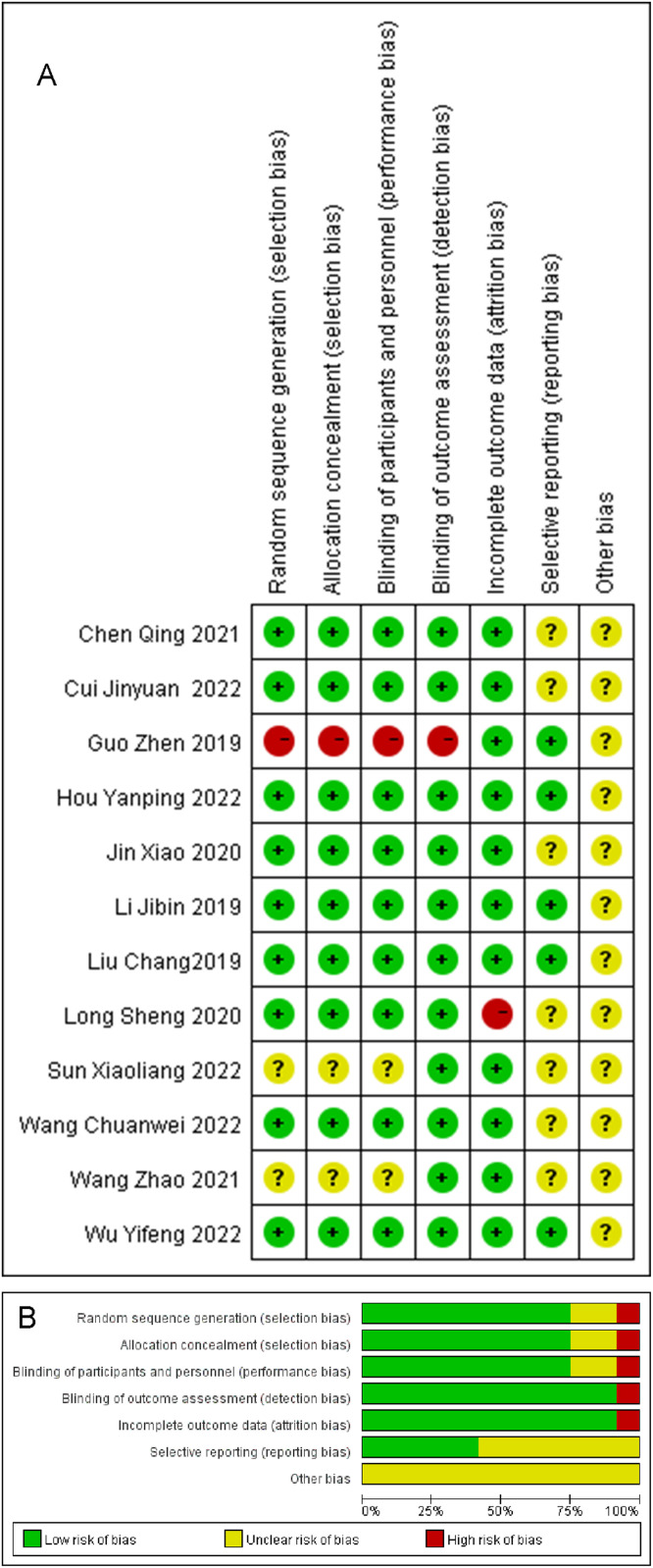
Risk of bias analysis (A) and percentage risk of bias (B) graphs.

The intricate details of these assessments, including individual risk evaluations for each study, are depicted in Fig. 2.

## Meta-analysis results

### *Effectiveness*

**Fig. 3:**
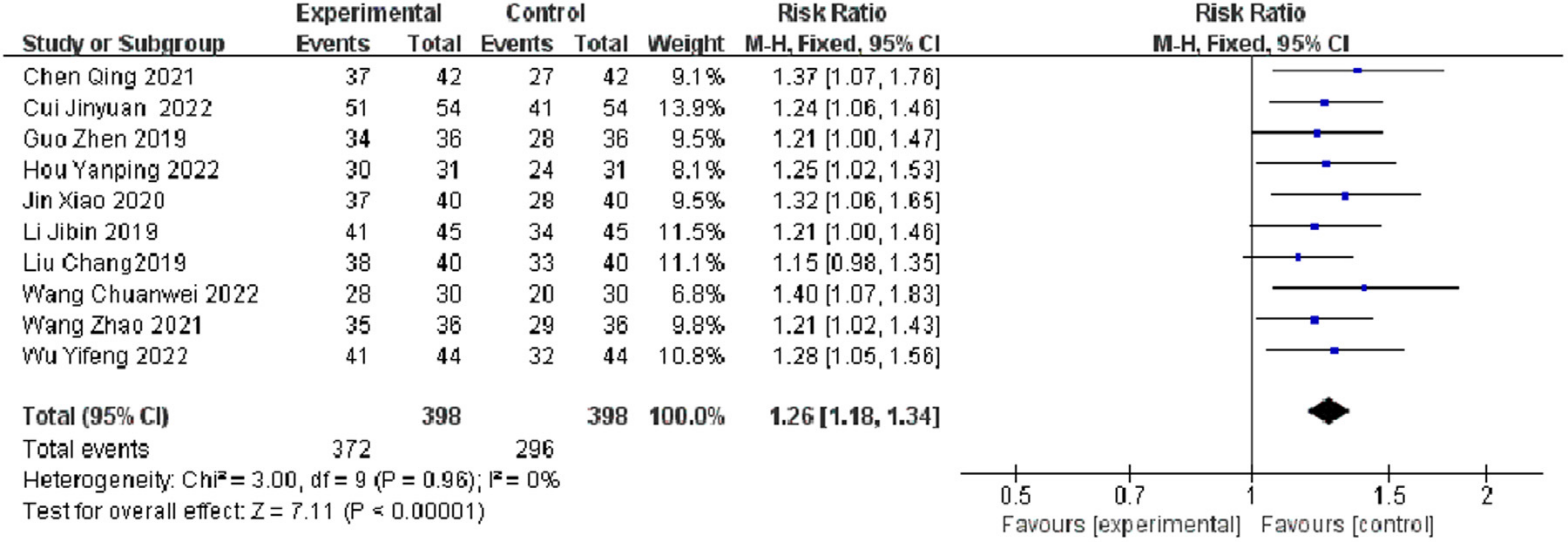
Comparison of the results of the observation group and the control group in terms of effective rate.

Ten Randomized Controlled Trials (RCTs) reported on the overall effectiveness rate. The analysis indicated no significant heterogeneity (*p* < 0.0001, I^2^ = 0 %). The observation group, receiving the combination treatment, showed a statistically significant difference compared to the control group in terms of the overall effectiveness rate (I^2^ = 0 %, RR = 1.26, 95 % CI [1.18, 1.34], *p* < 0.0001). This suggests that the observation group was superior to the control group concerning the total effective rate in treating migraine patients (Fig. 3).

### *Recurrence rate*

#### Overview

The recurrence rate was evaluated in 2 RCT studies.

#### Heterogeneity test results

The results indicated no heterogeneity (p = 0.003, I^2^ = 0 %).

#### Comparison between Groups

**Fig. 4:**
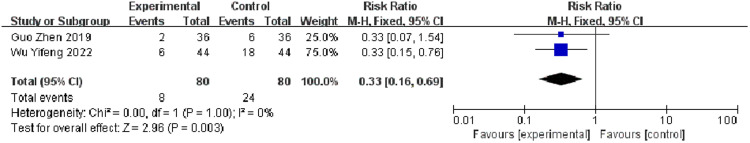
Comparison of the results of the recurrence rate between the observation group and the control group.

There was a statistically significant difference between the observation and control groups regarding the recurrence rate of migraines (I^2^ = 0 %, RR = 0.33, 95 % CI [0.16, 0.69], p = 0.003). The recurrence rate in the observation group was significantly lower than in the control group, indicating a substantial reduction in recurrence. However, the certainty of these results is limited due to the small number of articles included (Fig. 4).

### *TCM symptom score*

**Fig. 5:**
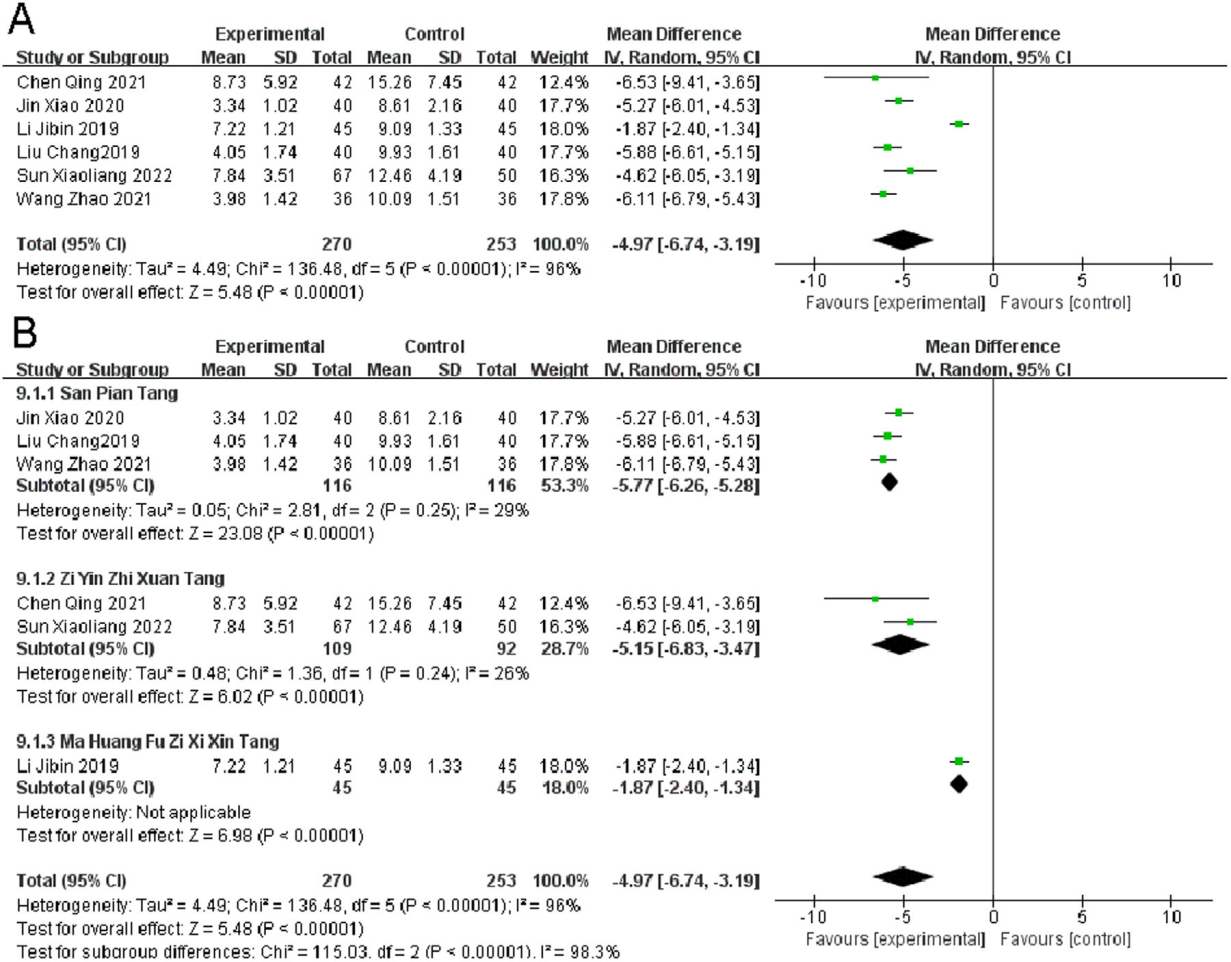
Comparison of the results of TCM symptom score (A) and subgroup analysis of TCM symptom score (B) between the observation group and the control group.

Six Randomized Controlled Trials (RCTs) reported on the TCM symptom score in migraine patients. Heterogeneity Test Results: Significant heterogeneity was observed (*p* < 0.00001, I^2^ = 96 %). Despite the heterogeneity, the observation group, when compared to the control group, showed a significant improvement in TCM symptom scores (I^2^ = 96 %, MD = -4.97, 95 % CI [-6.74, -3.19], *p* < 0.00001) (Fig. 5).

### *Endothelin level*

**Fig. 6:**
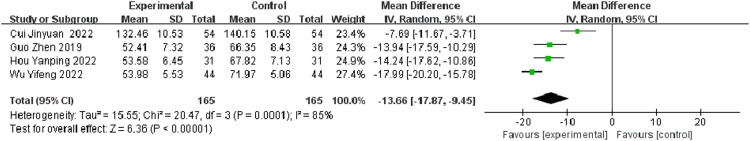
Comparison of the results of endothelin levels in the observation and control groups.

Four RCTs focused on endothelin levels in migraine patients. The analysis revealed significant heterogeneity (*p* < 0.00001, I^2^ = 85 %). The observation group demonstrated a statistically significant improvement in endothelin levels compared to the control group (I^2^ = 85 %, MD = -13.66, 95 % CI [-17.87, -9.45], p = 0.0001) (Fig. 6).

### *NRS scores*

**Fig. 7:**
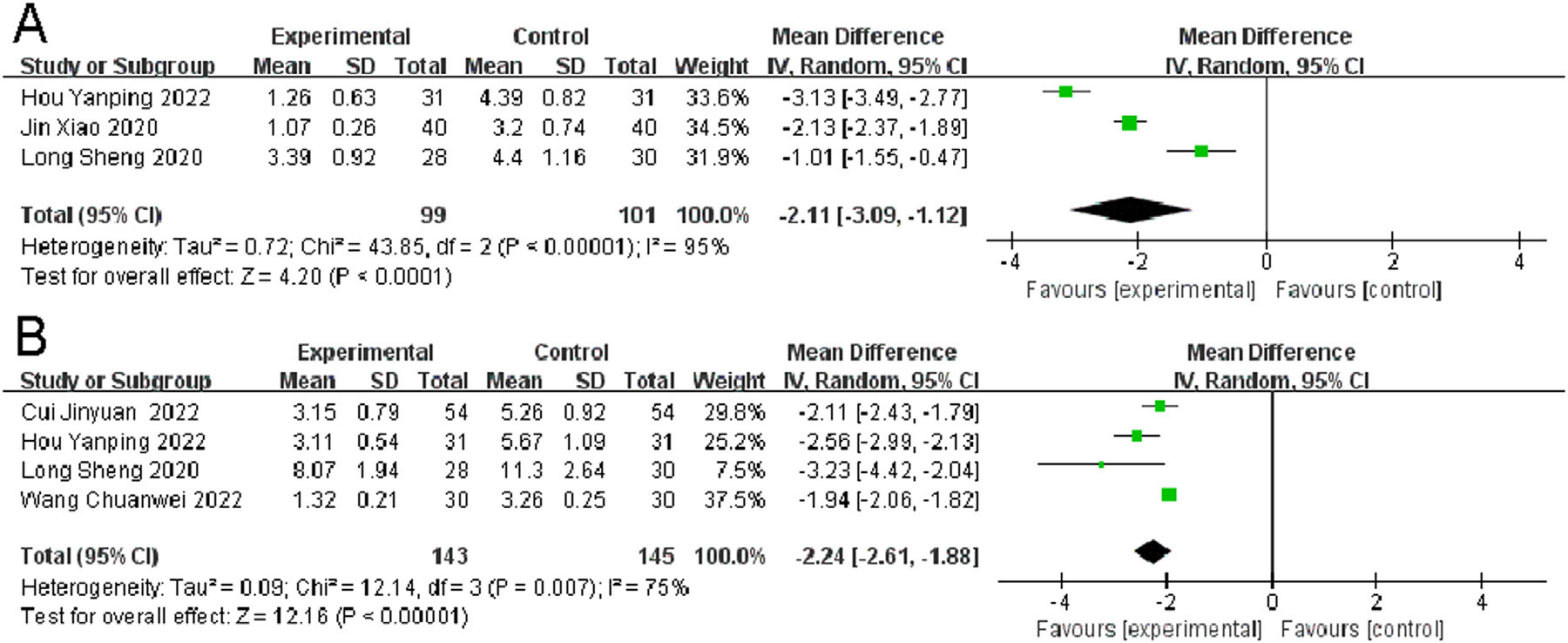
Comparison of the results of NRS scores (A) and VAS scores (B) between the observation and control groups.

Three RCTs examined NRS scores in migraine patients. There was significant heterogeneity found (*p* < 0.00001, I^2^ = 95 %). The observation group showed a significant reduction in NRS scores compared to the control group, indicating better outcomes (I^2^ = 95 %, MD = -2.11, 95 % CI [-3.09, -1.12], *p* < 0.0001) (Fig. 7A).

### *VAS scores*

Four Randomized Controlled Trials (RCTs) reported on VAS (Visual Analogue Scale) scores in migraine patients. The analysis showed significant heterogeneity (*p* < 0.00001, I^2^ = 75 %). The observation group exhibited a significantly better outcome in terms of change in VAS scores compared to the control group (I^2^ = 75 %, MD = -2.24, 95 % CI [-2.61, -1.88], p = 0.007) (Fig. 7B).

### *Number of episodes*

**Fig. 8:**
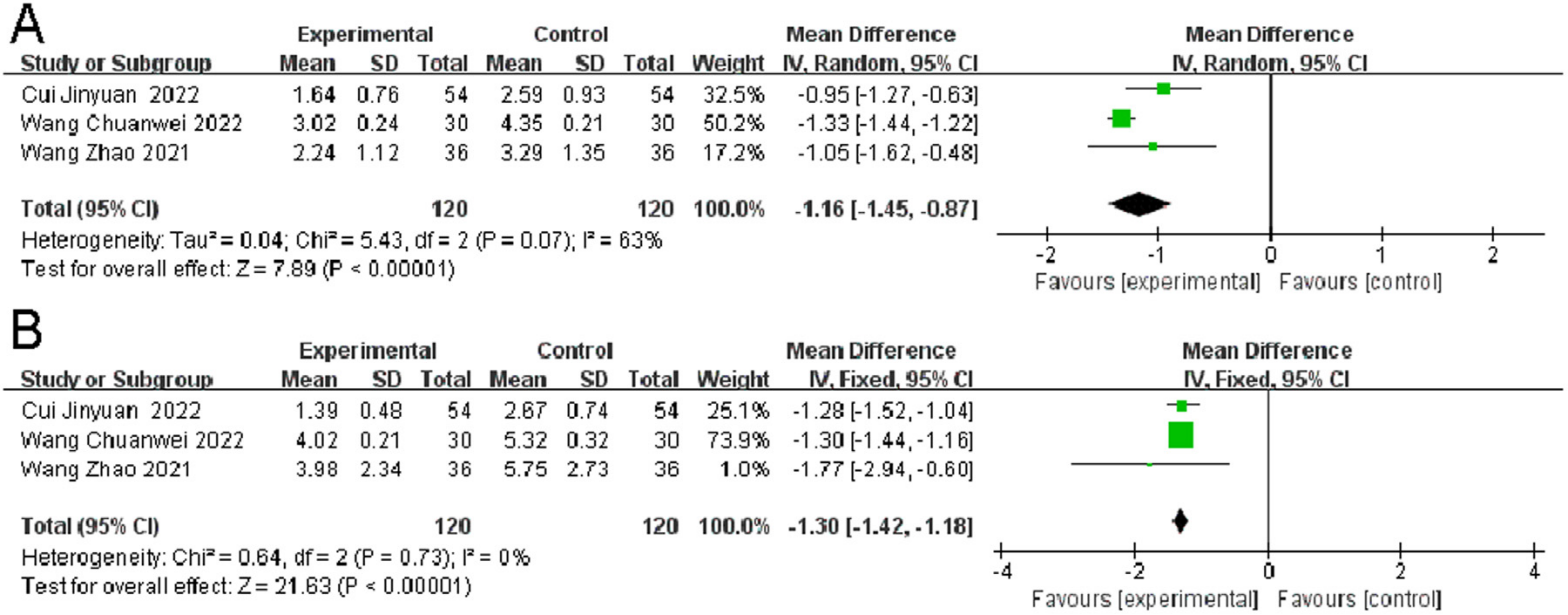
Comparison of the results of the number of episodes (A) and duration (B) between the observation and control groups.

Three RCTs assessed the number of migraine episodes. Significant heterogeneity was noted (*p* < 0.00001, I^2^ = 63 %). The observation group was superior to the control group in terms of the reduction in the number of episodes (I^2^ = 63 %, MD = -1.16, 95 % CI [-1.45, -0.87], p = 0.007). (Fig. 8A).

### Duration

Three RCTs reported on the duration of migraines. No significant heterogeneity was found (p = 0.73, I^2^ = 0 %). The difference between the observation and control groups was statistically non-significant regarding the duration of migraines (I^2^ = 0 %, MD = -1.30, 95 % CI [-1.42, -1.18], p = 0.73). The small number of studies and sample size were considered as influencing factors (Fig. 8B).

### *Publication bias*

**Fig. 9:**
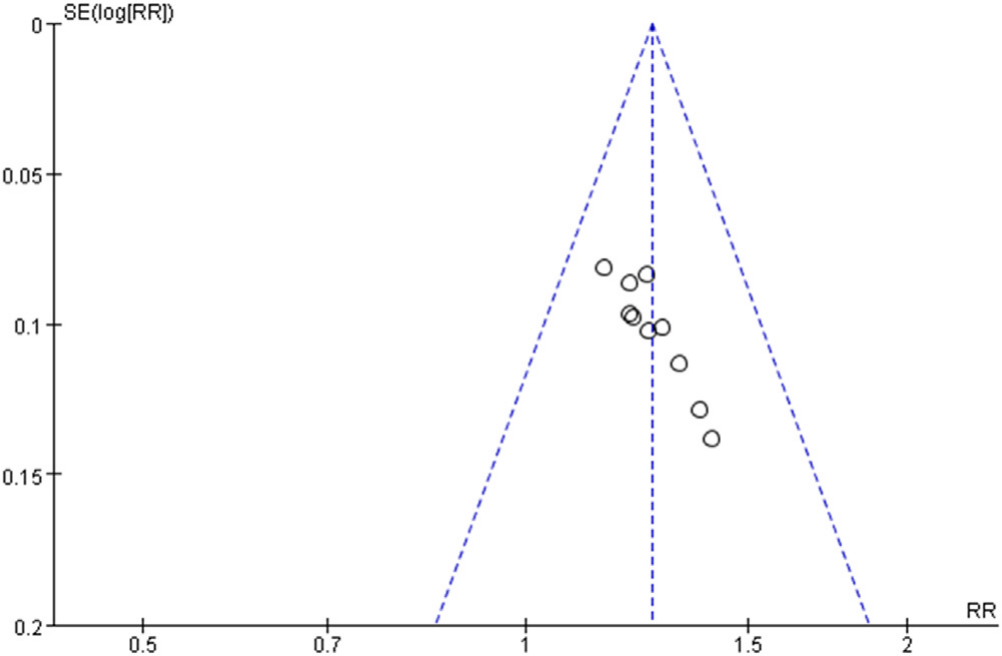
Funnel plot of the efficiency of migraine patients.

The bias analysis was conducted based on the efficiency of migraine patients. The MD (Mean Difference) value of each study was plotted as the horizontal coordinate against the inverse of the log standard error of the MD value as the vertical coordinate, creating a biased funnel plot. A test for publication bias was conducted on the included studies, and the results indicated asymmetry in the funnel plot, suggesting a certain degree of publication bias in the included literature (Fig. 9).

## Discussion

Migraine, a complex polygenic disorder, is characterized by recurrent headaches with neurovascular pathophysiological features. These attacks manifest as unilateral or bilateral throbbing headaches, predominantly on the hemianopic side of the head, and are often accompanied by photosensitivity, phonophobia, nausea, and vomiting. The precise etiology of migraine remains elusive; however, it is widely believed among experts and scholars that its occurrence is intricately linked to cerebral dysfunction and secondary vascular lesions. Wolff's 1963 hypothesis posited that abnormal vascular constriction might be a primary cause of migraine attacks.^16^ Subsequent research has suggested that neuronal activity and inflammatory mediators play significant roles.^17^^,^^18^ Studies have also demonstrated that factors such as excessive fatigue, mood disturbances, insufficient sleep, and overconsumption of alcohol can precipitate migraines.^19^

In clinical practice, Flunarizine Hydrochloride is a preferred pharmacological agent for migraine management. As a calcium channel antagonist, Flunarizine inhibits calcium channels, alleviates smooth muscle spasms, and reduces sensory organ sensitivity to migraine.^20^ Additionally, it enhances intracranial microcirculation and dilates blood vessels, thereby mitigating symptoms such as headache and vertigo.^21^ However, the therapeutic efficacy of Flunarizine monotherapy is limited, and its use is associated with notable adverse effects.

Traditional Chinese Medicine (TCM) categorizes migraine under the umbrella of “headache” disorders. TCM attributes headaches to imbalances in elements such as wind, fire, cold, phlegm, dampness, and stasis, with a particular emphasis on spleen deficiency, phlegm turbidity, blood stasis, and obstruction of collaterals leading to a deprivation of nourishment in the clear orifices. An example of a TCM remedy is MahuangFuzi Xixin decoction, derived from the “Treatise on Typhoid Fever”. This formula comprises Radix Pseudostellariae to promote Yang and disperse cold, Ephedra for epidermal cold relief and menstrual promotion, Sinapis for warming and pain relief, and Rhizoma Ligustici Chuanxiong and Radix Angelicae Sinensis to enhance blood circulation and alleviate pain.

The concept of “SanPian decoction” first appeared in “Bian Zheng Lu: Headache Section”. This formula includes ingredients such as Chuanxiong, Angelica dahurica, white mustard seed, Cyperus rotundus, Bupleurum, Prunus mume seed, and licorice. It is utilized to soothe the liver, alleviate depression, promote blood circulation, remove blood stasis, and relieve pain.

In this research, a meta-analysis encompassing 12 domestic and international clinical studies with a total of 973 migraine participants was conducted to evaluate the efficacy and safety of Flunarizine Hydrochloride combined with Chinese herbal decoction. The analysis revealed that this combination therapy notably enhances the management of migraine, effectively improving TCM evidence scores, endothelin levels, NRS scores, VAS scores, the frequency of headache episodes, and the duration of headaches.

However, the results of this study are subject to certain limitations. Primarily, the scope of the included literature was relatively narrow. The language restriction to Chinese and English may have contributed to a limited literature pool, potentially affecting the breadth of the analysis. Additionally, the risk of bias assessment in the included studies indicated a predominant high-risk bias. For instance, achieving genuine blinding in trials proved challenging, as most participants were likely aware of the treatment process and related precautions, considering both the study design and the prevailing medical environment.

Moreover, the specific focus on migraine sufferers in the included literature might have led to issues with poor adherence. Thus, future Randomized Controlled Trials (RCTs) should endeavor to standardize trial designs to mitigate bias risk factors. In terms of outcome observation, more objective data, such as hemodynamic and serological indicators, should be considered. These changes would align with international guidelines for migraine prevention trials, which recommend specific outcome measures like the change in the number of migraine days, the alteration in migraine severity, and the maintenance rate of 50 percent migraine relief.^22^ Comprehensive documentation of missed visits and adverse events is also essential to enhance the scientific rigor of the study.

## Conclusion

Further research, employing standardized protocols and consistent outcome measures, is crucial to deepen the understanding of TCM's role in migraine management. Future meta-analyses incorporating larger, more diverse, and high-quality RCTs will be invaluable in expanding clinical treatment options for migraine.

### Authors' contributions

Dan Fan wrote and reviewed the draft. Wei Leng and Liqin Zhang collected the data and performed the statistical analysis. All authors read and approved the final manuscript.

## Funding

This research did not receive any specific grant from funding agencies in the public, commercial, or not-for-profit sectors.

## Conflicts of interest

The authors declare no conflicts of interest.
